# Supplementary material for: Mechanisms of school-based peer education interventions to improve young people’s health literacy or health behaviours: A realist-informed systematic review
Source: PLoS One. 2024 May 31;19(5):e0302431. doi: 10.1371/journal.pone.0302431 (PMC11142678; doi:10.1371/journal.pone.0302431)
Supplement: S2 Table — (DOCX) [file pone.0302431.s002.docx]

**S2 Table: Full Search Strategy**

|  | **Academic Search Complete** | **CINAHL** | **Embase** | **ERIC** | **MEDLINE** | **PsycInfo** |  |
| --- | --- | --- | --- | --- | --- | --- | --- |
| **Search terms pertaining to schools and school students** | ( DE "SCHOOLS" OR DE "SCHOOL children" OR DE "HIGH school students" OR DE "MIDDLE school students" OR DE "PRIMARY schools" OR DE "PRIMARY education" OR DE "SECONDARY education" OR DE "SECONDARY schools" OR DE "ELEMENTARY schools" ) OR TI ( "Elementary School*" OR "Primary School*" OR "Junior School*" OR "Junior High School" OR "Secondary School*" OR "Middle School*" OR "Sixth Form*" OR "High School*" OR "Primary education" OR "Secondary education" OR "Special Education*" OR "School-based" OR "school based" ) OR AB ("Elementary School*" OR "Primary School*" OR "Junior School*" OR "Junior High School" OR "Secondary School*" OR "Middle School*" OR "Sixth Form*" OR "High School*" OR "Primary education" OR "Secondary education" OR "Special Education*" OR "School-based" OR "school based" ) | MH ( "Students, High School" OR "Students, Middle School" OR "Students, Elementary" OR "Schools, Secondary" OR "Schools, Middle" OR "Schools" ) OR TI ( "Elementary School*" OR "Primary School*" OR "Junior School*" OR "Junior High School" OR "Secondary School*" OR "Middle School*" OR "Sixth Form*" OR "High School*" OR "Primary education" OR "Secondary education" OR "Special Education*" OR "School-based" OR "school based" ) OR AB ( "Elementary School*" OR "Primary School*" OR "Junior School*" OR "Junior High School" OR "Secondary School*" OR "Middle School*" OR "Sixth Form*" OR "High School*" OR "Primary education" OR "Secondary education" OR "Special Education*" OR "School-based" OR "school based" ) | (school or school child or high school student or high school or middle school or middle school student or elementary student or primary school).sh. or ("Elementary School*" or "Primary School*" or "Junior School*" or "Junior High School" or "Secondary School*" or "Middle School*" or "Sixth Form*" or "High School*" or "Primary education" or "Secondary education" or "Special Education*" or "School-based" or "school based").ti. or ("Elementary School*" or "Primary School*" or "Junior School*" or "Junior High School" or "Secondary School*" or "Middle School*" or "Sixth Form*" or "High School*" or "Primary education" or "Secondary education" or "Special Education*" or "School-based" or "school based").ab. | N/A - ERIC is an educational database | ( MH "Schools" ) OR TI ( "Elementary School*" OR "Primary School*" OR "Junior School*" OR "Junior High School" OR "Secondary School*" OR "Middle School*" OR "Sixth Form*" OR "High School*" OR "Primary education" OR "Secondary education" OR "Special Education*" OR "School-based" OR "school based" ) OR AB ( "Elementary School*" OR "Primary School*" OR "Junior School*" OR "Junior High School" OR "Secondary School*" OR "Middle School*" OR "Sixth Form*" OR "High School*" OR "Primary education" OR "Secondary education" OR "Special Education*" OR "School-based" OR "school based" ) | ( DE "School Learning" OR DE "Classmates" OR DE "Elementary Education" OR DE "High School Education" OR DE "Middle School Education" OR DE "Secondary Education" OR DE "Elementary Schools" OR DE "High Schools" OR DE "Junior High Schools" OR DE "Middle Schools" OR DE "Elementary School Students" OR DE "High School Students" OR DE "Junior High School Students" OR DE "Middle School Students") OR TI ( "elementary student*" OR "high school student*" OR "middle school student*" OR "School*" OR "Sixth Form*" or "Special Education*" ) OR AB ( "elementary student*" OR "high school student*" OR "middle school student*" OR "School*" OR "Sixth Form*" or "Special Education*" ) |  |
| **Search terms pertaining to peer education** | DE "Peer teaching" OR TI ( peer educat*" OR "peer-led" OR "Peer led" OR "Student-led" OR "Student led" OR "Peer Teach*" OR "Peer Intervention* OR "Cross-age" or "Cross age" or "Peer to Peer" or "Peer-to-Peer" OR "Peer-delivered" or "Peer Delivered" ) OR AB ( peer educat*" OR "peer-led" OR "Peer led" OR "Student-led" OR "Student led" OR "peer teact*" OR "Peer Intervention* OR "Cross-age" or "Cross age" or "Peer to Peer" or "Peer-to-Peer" OR "Peer-delivered" or "Peer Delivered" ) | TI ( "peer educat*" OR "peer-led" OR "Peer led" OR "Student-led" OR "Student led" OR "Peer teach*" OR "Peer Intervention*" OR "Cross-age" or "Cross age" or "Peer to Peer" or "Peer-to-Peer" OR "Peer-delivered" or "Peer Delivered" ) OR AB ( "peer educat*" OR "peer-led" OR "Peer led" OR "Student-led" OR "Student led" OR "Peer teach*" OR "Peer Intervention*" OR "Cross-age" or "Cross age" or "Peer to Peer" or "Peer-to-Peer" OR "Peer-delivered" or "Peer Delivered" ) | ("peer educat*" or "peer-led" or "Peer led" or "Student-led" or "Student led" or "Peer teach*" or "Peer Intervention*" or "Cross-age" or "Cross age" or "Peer to Peer" or "Peer-to-Peer" or "Peer-delivered" or "Peer Delivered").ti. or ("peer educat*" or "peer-led" or "Peer led" or "Student-led" or "Student led" or "Peer teach*" or "Peer Intervention*" or "Cross-age" or "Cross age" or "Peer to Peer" or "Peer-to-Peer" or "Peer-delivered" or "Peer Delivered").ab. | DE ( ""Peer Teaching" ) OR TI ( "peer educat*" OR "peer-led" OR "Peer led" OR "Student-led" OR "Student led" OR "Peer Teach*" OR "Peer Intervention* OR "Cross-age" or "Cross age" or "Peer to Peer" or "Peer-to-Peer" OR "Peer-delivered" or "Peer Delivered" ) OR AB ( "peer educat*" OR "peer-led" OR "Peer led" OR "Student-led" OR "Student led" OR "Peer teach*" OR "Peer Intervention* OR "Cross-age" or "Cross age" or "Peer to Peer" or "Peer-to-Peer" OR "Peer-delivered" or "Peer Delivered" ) | TI ( "peer educat*" OR "peer-led" OR "Peer led" OR "Student-led" OR "Student led" OR "Peer teach*" OR "Peer Intervention* OR "Cross-age" or "Cross age" or "Peer to Peer" or "Peer-to-Peer" OR "Peer-delivered" or "Peer Delivered" ) OR AB ( "peer educat*" OR "peer-led" OR "PEER LED" OR "STUDENT-LED" OR "STUDENT LED" OR "PEER TEACH*" OR "Peer Intervention* OR "Cross-age" or "Cross age" or "Peer to Peer" or "Peer-to-Peer" OR "Peer-delivered" or "Peer Delivered" ) | DE "Peer Tutoring" OR TI ( "peer educat*" OR "peer-led" OR "Peer led" OR "Student-led" OR "Student led" OR "Peer teach*" OR "Peer Intervention* OR "Cross-age" or "Cross age" or "Peer to Peer" or "Peer-to-Peer" OR "Peer-delivered" or "Peer Delivered" ) OR AB ( "peer educat*" OR "peer-led" OR "PEER LED" OR "STUDENT-LED" OR "STUDENT LED" OR "PEER TEACH*" OR "Peer Intervention* OR "Cross-age" or "Cross age" or "Peer to Peer" or "Peer-to-Peer" OR "Peer-delivered" or "Peer Delivered" ) |  |
| **Search terms pertaining to health-related interventions, health-related outcome domains** | ( DE "HEALTH education" OR DE "MENTAL health education" OR DE "HEALTH literacy" OR DE "HEALTH promotion" OR DE "HEALTH programs" OR DE "HEALTH promotion -- Social aspects" OR DE "DRUG abuse education" OR DE "DRUG abuse education in middle schools" OR DE "DRUG abuse education in secondary schools" OR DE "YOUTH & drugs" OR DE "DRUG abuse education in secondary schools" OR DE "YOUTH -- Substance use" OR DE "SUBSTANCE abuse prevention" OR DE "SUBSTANCE abuse" OR DE "YOUTH -- Tobacco use" OR DE "SMOKING" OR DE "ALCOHOLISM education” OR DE "ALCOHOLISM education" OR DE "YOUTH & alcohol" OR DE "SEX education" OR DE "SEX education for boys" OR DE "SEX education for children" OR DE "SEX education for girls" OR DE "SEX education for teenagers" OR DE "SAFE sex" OR DE "MENTAL health" OR DE "MENTAL health education" OR DE "CONFIDENCE" OR DE "CONFIDENCE in children" OR DE "MENTAL depression" OR DE "DEPRESSION in adolescence" OR DE "DEPRESSION in children" OR DE "ANXIETY" OR DE "ANXIETY in adolescence" OR DE "ANXIETY in children” OR DE "EMOTIONAL competence" OR DE "EMOTIONAL intelligence" OR DE "RISK-taking behavior" OR DE "RISK-taking behavior in adolescence" OR DE "RISK-taking behavior in children" OR DE "SELF-esteem in children" ) OR TI ( “Health Promotion" OR "Health Literacy" OR "health education" OR "School Based Intervention*" OR "Group Intervention*" OR “Smoking” OR “Drug abuse” OR “Substance abuse” OR “Drug education” OR "alcohol*" OR “Mental health” OR “Esteem” OR “Confidence” OR “Emotional” OR “behaviour*” OR “Depression” or “anxiety” OR “Sex education" OR "Safe sex" OR “RISK TAKING” OR “HEALTH RISK*” ) OR AB ( "Health Promotion" OR "Health Literacy" OR "health education" OR "School Based Intervention*" OR "Group Intervention*" OR “Smoking” OR “Drug abuse” OR “Substance abuse” OR “Drug education” OR "alcohol*" OR “Mental health” OR “Esteem” OR “Confidence” OR “Emotional” OR “behaviour*” OR “Depression” or “anxiety” OR “Sex education" OR "Safe sex" OR “RISK TAKING” OR “HEALTH RISK*” ) | MH ( "Health Education+" OR "Health Literacy+" OR "Health Promotion” OR "Health Information" OR "Mental Health" OR "Community Mental Health Services" OR "Confidence" OR "Emotional Regulation" OR "Emotional Maturity" OR "Emotional Intelligence" OR "Depression" OR "Anxiety" OR "Self Concept" OR "Child Behavior" OR "Adolescent Behavior" OR "Impulsive Behavior" OR "Risk Taking Behavior" OR "Substance Abuse" OR "Alcohol Abuse" OR “Street Drugs" OR "Behavior, Addictive" OR "Alcohol Abuse" OR "Smoking" OR "Sexual Health" OR "Safe Sex" OR "Sex Education" ) OR TI (“Health Promotion" OR "Health Literacy" OR "health education" OR "School Based Intervention*" OR "Group Intervention*" OR “Smoking” OR “Drug abuse” OR “Substance abuse” OR “Drug education” OR "alcohol*" OR “Mental health” OR “Esteem” OR “Confidence” OR “Emotional” OR “behaviour*” OR “Depression” or “anxiety” OR “Sex education" OR "Safe sex" OR “RISK TAKING” OR “HEALTH RISK*” ) OR AB ( “Health Promotion" OR "Health Literacy" OR "health education" OR "School Based Intervention*" OR "Group Intervention*" OR “Smoking” OR “Drug abuse” OR “Substance abuse” OR “Drug education” OR "alcohol*" OR “Mental health” OR “Esteem” OR “Confidence” OR “Emotional” OR “behaviour*” OR “Depression” or “anxiety” OR “Sex education" OR "Safe sex" OR “RISK TAKING” OR “HEALTH RISK*” ) | (health promotion or health program or mental health service or health education or health literacy or school mental health service or school health education or school health service or mental health or anxiety or depression or adolescent depression or behavior control or behavior change or coping behavior or emotional intelligence or emotion or self concept or self esteem or substance abuse or drug abuse or adolescent smoking or smoking or smoking reduction or smoking prevention or alcohol abuse or addiction or alcohol consumption or safe sex or sexual education or high risk behavior).sh. or ("Health Promotion" or "Health Literacy" or "health education" or "School Based Intervention*" or "Group Intervention*" or "Smoking" or "Drug abuse" or "Substance abuse" or "Drug education" or "Alcohol*" or "Mental health" or "Esteem" or "Confidence" or "Emotional" or "behaviour*" or "Depression" or "anxiety" or "Sex education" or "Safe sex" or "RISK TAKING" or "HEALTH RISK*").ti. or ("Health Promotion" or "Health Literacy" or "health education" or "School Based Intervention*" or "Group Intervention*" or "Smoking" or "Drug abuse" or "Substance abuse" or "Drug education" or "Alcohol*" or "Mental health" or "Esteem" or "Confidence" or "Emotional" or "behaviour*" or "Depression" or "anxiety" or "Sex education" or "Safe sex" or "RISK TAKING" or "HEALTH RISK*").ab. | DE ( "Health Promotion" OR "Health Education" OR "Comprehensive School Health Education" OR "Health Programs" OR "Self Control" OR "Mental Health Programs" OR "Mental Health" OR "Self Destructive Behavior" OR "Depression (Psychology)" OR "Anxiety" OR "Emotional Development" OR "Behavior Change" OR "Alcohol Education" OR "Drug Education" OR "Drug Abuse" OR "Drug Addiction" OR "Substance Abuse" OR "Smoking" OR "Sex Education" OR "Behavior Change" OR "Attitude Change" ) OR TI ( “Health Promotion" OR "Health Literacy" OR "health education" OR "School Based Intervention*" OR "Group Intervention*" OR “Smoking” OR “Drug abuse” OR “Substance abuse” OR “Drug education” OR "Alcohol*" OR “Mental health” OR “Esteem” OR “Confidence” OR “Emotional” OR “behaviour*” OR “Depression” or “anxiety” OR “Sex education" OR "Safe sex" OR “RISK TAKING” OR “HEALTH RISK*” ) OR AB ( "Health Promotion" OR "Health Literacy" OR "health education" OR "School Based Intervention*" OR "Group Intervention*" OR “Smoking” OR “Drug abuse” OR “Substance abuse” OR “Drug education” OR "Alcohol*" OR “Mental health” OR “Esteem” OR “Confidence” OR “Emotional” OR “behaviour*” OR “Depression” or “anxiety” OR “Sex education" OR "Safe sex" OR “RISK TAKING” OR “HEALTH RISK*” ) | MH ( "Health Education+" OR "Health Literacy+" OR “Health promotion” OR "School Health Services" OR "Preventive Health Services" OR “School Mental Health Services" OR "Anxiety" OR "Self Concept" OR "Depression” OR “Emotional Intelligence" OR “Psychological Distress” OR “Adaptation, Psychological" OR "Sex Education" OR "Sexual Behavior" OR "Safe Sex" OR "Tobacco Use" OR "Underage Drinking" OR "Alcoholism" OR "Substance-Related Disorders" OR "Substance Abuse, Intravenous” OR "Risk Reduction Behavior" OR "Impulsive Behavior" OR "Risk-Taking” OR “Health Risk Behaviors” OR "Health Behavior" ) OR TI ( “Health Promotion" OR "Health Literacy" OR "health education" OR "School Based Intervention*" OR "Group Intervention*" OR “Smoking” OR “Drug abuse” OR “Substance abuse” OR “Drug education” OR "alcohol*" OR “Mental health” OR “Esteem” OR “Confidence” OR “Emotional” OR “behaviour*” OR “Depression” or “anxiety” OR “Sex education" OR "Safe sex" OR “RISK TAKING” OR “HEALTH RISK*” ) OR AB ( "Health Promotion" OR "Health Literacy" OR "health education" OR "School Based Intervention*" OR "Group Intervention*" OR “Smoking” OR “Drug abuse” OR “Substance abuse” OR “Drug education” OR "alcohol*" OR “Mental health” OR “Esteem” OR “Confidence” OR “Emotional” OR “behaviour*” OR “Depression” or “anxiety” OR “Sex education" OR "Safe sex" OR “RISK TAKING” OR “HEALTH RISK*” ) | ( DE "School Based Intervention" OR DE "Health Promotion" OR DE "Health Knowledge" OR DE "Health Literacy" OR DE "Health Education" OR DE "Health Information" OR DE "Public Health Campaigns" OR DE "Preventive Health Services" OR DE "Mental Health Literacy" OR DE "Mental Health Program Evaluation" OR "Preventive Mental Health Services" OR DE "Self-Confidence" OR DE "Self-Esteem" OR DE "Health Risk Behavior” OR DE "Anxiety" OR DE "Depression (Emotion)" OR DE "Emotional Control" OR DE "Behavior Change" OR DE "Drug Education" OR DE "Substance Use Prevention" OR DE "Substance Use Treatment" OR DE "Harm Reduction" OR DE "Underage Drinking" OR DE "Tobacco Smoking" OR DE "Sexual Risk Taking” OR DE "Sex Education" OR DE "Safe Sex" ) OR TI ( “Health Promotion" OR "Health Literacy" OR "health education" OR "School Based Intervention*" OR "Group Intervention*" OR “Smoking” OR “Drug abuse” OR “Substance abuse” OR “Drug education” OR "Alcohol*" OR “Mental health” OR “Esteem” OR “Confidence” OR “Emotional” OR “behaviour*” OR “Depression” or “anxiety” OR “Sex education" OR "Safe sex" OR “RISK TAKING” OR “HEALTH RISK*” ) OR AB ( "Health Promotion" OR "Health Literacy" OR "health education" OR "School Based Intervention*" OR "Group Intervention*" OR “Smoking” OR “Drug abuse” OR “Substance abuse” OR “Drug education” OR "Alcohol*" OR “Mental health” OR “Esteem” OR “Confidence” OR “Emotional” OR “behaviour*” OR “Depression” or “anxiety” OR “Sex education" OR "Safe sex" OR “RISK TAKING” OR “HEALTH RISK*” ) |  |
| **Updated hits 12/10/2020 (no limiters, search terms amended)** | **108** | **120** | **567** | **963** | **137** | **230** |  |
