# Supplementary material for: Mechanisms of school-based peer education interventions to improve young people’s health literacy or health behaviours: A realist-informed systematic review
Source: PLoS One. 2024 May 31;19(5):e0302431. doi: 10.1371/journal.pone.0302431 (PMC11142678; doi:10.1371/journal.pone.0302431)
Supplement: S3 Table — (DOCX) [file pone.0302431.s003.docx]

**S3 Table: Overview of Included Studies**

| Author, year location | Health Area | Health Category | Sample Size | Study Design | Mechanisms | Identification of mechanisms | Quality  Score | Quality  Rating | Use of logic model or programme theory? (Y/N) | Reference to intervention 'mechanisms' (Y/N) |
| --- | --- | --- | --- | --- | --- | --- | --- | --- | --- | --- |
| Alcohol, smoking, substance use | | | | | | | | | | |
| Al-Sheyab et al. 2016 USA | Smoking prevention | Alcohol, smoking, substance use | 486 | Quantitative; cluster RCT | 1) School values and broader change in school culture 2) Frequency of interaction and informal diffusion beyond the classroom 3) Student nominated peers and peers as role models | Author reflections in discussion | 2 | Medium | N | N |
| Audrey et al. 2006 UK | Smoking prevention | Alcohol, smoking, substance use | 10,730 | Qualitative process evaluation | 1) Peerness; similar, relatable and credible 2) Informal, innovative and personalised delivery methods 3) Friendship group and closeness to a peer educator 4) Frequency of interaction and informal diffusion beyond the classroom | Qualitative data; peer educator interviews and peer learner focus groups | 4 | High | N | N |
| Audrey et al. 2008 UK | Smoking prevention | Alcohol, smoking, substance use | 59 schools | Qualitative process Evaluation (teachers perspectives) | 1) A balance between autonomy and support 2) Peerness; similar, relatable and credible 3) Safe space – non-judgemental space to share experiences 4) Student nominated peers and peers as role models | Qualitative data; teacher interviews | 4 | High | N | N |
| Bloor et al. 1999 UK, Wales | Smoking prevention | Alcohol, smoking, substance use | 1247 | Quantitative; controlled experimental design | 1) Friendship groups & closeness to a peer educator | Author reflections in discussion | 1 | Low | N | N |
| Campbell et al. 2008 UK | Smoking prevention | Alcohol, smoking, substance use | 10,730 | Quantitative; Cluster randomised controlled trial | 1) A balance between autonomy and support 2) Friendship group and closeness to a peer educator | Quantitative data and author reflections in discussion | 5 | High | N | N |
| Demirezen et al. 2020 Turkey | Substance abuse | Alcohol, smoking, substance use | 663 | Quantitative; pre-test/post-test | 1) Informal, innovative and personalised delivery methods 2) Safe space – non-judgemental space to share experiences | Quantitative data and author reflections in discussion | 3 | Medium | N | N |
| Dobbie et al. 2019 UK | Smoking prevention | Alcohol, smoking, substance use | 2491 | Mixed methods process evaluation | 1) School values and broader change in school culture 2) Peerness; similar, relatable and credible 3) Friendship groups & closeness to a peer educator | Qualitative data; staff and student interviews and focus groups | 2 | Medium | N | Y |
| LaChause & Robert, 2008 USA | Preventing fetal alcohol syndrome | Alcohol, smoking, substance use | 114 | Quantitative; longitudinal quasi-experimental design | Frequency of interaction  Personalised/dyanmic delivery (lack of!) | Author reflections in discussion | 1 | Low | N | N |
| Nurmala et al. 2020 Indonesia | Drug abuse prevention | Alcohol, smoking, substance use | 10 | Qualitative case study | 1) A balance between autonomy and support 2) Peerness; similar, relatable and credible 3) Friendship groups & closeness to a peer educator 4) Safe space – non-judgemental space to share experiences | Qualitative data; peer educator interviews | 3 | Medium | N | N |
| Perry et al. 1980 USA | Smoking prevention | Alcohol, smoking, substance use | 707 | Quantitative; controlled trial | 1) School values and broader change in school culture | Author reflections in discussion | 4 | High | N | N |
| Shah et al. 2001 Australia | Asthma and smoking prevention | Alcohol, smoking, substance use | 272 | Quantitative; cluster randomised controlled trial | 1) Peerness; similar, relatable and credible 2) Student nominated peers and peers as role models | Quantitative data and author reflections in discussion | 3 | Medium | N | N |
| Starkey et al. 2009 UK | Smoking prevention | Alcohol, smoking, substance use | 10,730 | Mixed methods; Cluster randomised controlled trial | 1) Peerness; similar, relatable and credible 2) Informal, innovative and personalised delivery methods 3) Friendship groups & closeness to a peer educator 4) Student nominated peers and peers as role models | Qualitative data; peer learner and peer educator interviews and focus groups, staff interviews | 3 | Medium | N | N |
| Weichold & Silbereisen, 2012 Germany | Alcohol and smoking prevention | Alcohol, smoking, substance use | 105 | Quantitative; randomised pilot trial | 1) A balance between autonomy and support 2) Peerness; similar, relatable and credible 3) Student nominated peers and peers as role models | Quantitative data and author reflections in discussion | 2 | Medium | N | Y |
| Health Lifestyles | | | | | | | | | | |
| Ajuwon & Ajuwon, 2019 Nigeria | Online health resources and health literacy | Healthy lifestyles | 120 | Mixed methods | 1) A balance between autonomy and support 2) Peerness; similar, relatable and credible 3) Frequency of interaction and informal diffusion beyond the classroom | Qualitative data; | 2 | Medium | N | N |
| Bell et al. 2017 UK | Obesity prevention | Healthy lifestyles | 928 | Mixed methods; exploratory trial | 1) A balance between autonomy and support 2) School values and broader change in school culture 3) Simplicity of health messages | Quantitative data, qualitative focus group data and author reflections in discussion | 4 | High | N | N |
| Bogart et al. 2014 USA | Nutrition and exercise | Healthy lifestyles | 1524 | Quantitative; randomised controlled trial | 1) School values and broader change in school culture | Author reflections in discussion | 2 | Medium | N | Y |
| Cui et al. 2012 China | Physical activity and sedentary behaviour | Healthy lifestyles | 682 | Quantitative; randomised controlled trial | 1) Peerness; similar, relatable and credible 2) Friendship groups & closeness to a peer educator | Quantitative data and author reflections in discussion | 3 | Medium | N | N |
| McQuinn et al. 2022 UK | Physical activity | Healthy lifestyles | 287 | Mixed methods | 1) Peerness; similar, relatable and credible 2) Friendship groups & closeness to a peer educator | Qualitative data | 4 | High | N | N |
| Meyer et al. 2000 USA | General health promotion | Healthy lifestyles | 796 | Mixed methods; program implementation evaluation | 1) School values and broader change in school culture | Quantitative data and author reflections in discussion | 1 | Low | N | N |
| Ping et al. 2014 China | Sun safety | Healthy lifestyles | 609 | Mixed methods; Cluster randomised controlled trial | 1) A balance between autonomy and support 2) Informal, innovative and personalised delivery methods 3) Peers as role models | Qualitative data; peer learner interviews | 4 | High | N | N |
| Sebire et al. 2019a UK (*Process evaluation*) | Physical activity | Healthy lifestyles | 52 | Mixed methods process evaluation | 1) A balance between autonomy and support  2) Peerness; similar, relatable and credible  3) Friendship groups & closeness to a peer educator  4) Student nominated peers and peers as role models  5) Frequency of interaction and informal diffusion beyond the classroom  6) Safe space – non-judgemental space to share experiences | Qualitative data; peer learner and peer educator focus groups | 4 | High | N | Y |
| Sebire et al. 2019b *(Feasibility RCT)* | Physical activity | Healthy lifestyles | 427 | Mixed methods; Cluster randomised controlled trial | 1) A balance between autonomy and support 2) School values and broader change in school culture | Qualitative data; peer learner and peer educator focus groups | 5 | High | Y | Y |
| Mental Health | | | | | | | | | | |
| Ellis, 2009 | Promoting self-concept | Mental health | 483 | Mixed-methods | 1) School values and broader change in school culture 2) Friendship groups & closeness to a peer educator | Qualitative data; peer learner and peer educator focus groups | 4 | High | N | N |
| Parikh et al. 2018 USA | Depression awareness | Mental health | 878 | Quantitative; pre-post questionnaire | 1) A balance between autonomy and support | Author reflections in discussion | 1 | Low | N | N |
| Pickering et al. 2018 USA | Suicide prevention | Mental health | 4263 | Quantitative social network analysis | 1) School values and broader change in school culture 2) Friendship groups & closeness to a peer educator 3) Frequency of interaction and informal diffusion beyond the classroom | Quantitative data; predictors of exposure | 5 | High | N | Y |
| Wright-Berryman et al. 2019 USA | Suicide prevention | Mental health | 1174 | Quantitative; descriptive pilot data | 1) School values and broader change in school culture 2) Friendship groups & closeness to a peer educator 3) Safe space – non-judgemental space to share experiences | Quantitative data and author reflections in discussion | 3 | Medium | N | N |
| Wyman et al. 2010 USA | Suicide prevention | Mental health | 3128 | Quantitative; randomised trial with waitlist control | 1) 1) A balance between autonomy and support 2) School values and broader change in school culture 3) Peerness; similar, relatable and credible 4) Ratio of peer educators to peer learners | Quantitative data (help-seeking attitudes) and author reflections in discussion | 4 | High | N | N |
| Sex Education | | | | | | | | | | |
| Caron et al. 2004 Canada | AIDS/STD awareness | Sex education | 1004 | Quantitative; quasi-experimental design | 1) A balance between autonomy and support 2) Informal, innovative and personalised delivery methods | Author reflections in discussion | 3 | Medium | N | N |
| Ito et al. 2022 Tanzania | Sexual health promotion | Sex education | 92 | Qualitative | 1) Peerness; similar, relatable and credible 2) Informal, innovative and personalised delivery methods 3) Friendship groups & closeness to a peer educator 4) Safe space – non-judgemental space to share experiences | Qualitative data; peer educator and peer learner focus groups | 3 | Medium | N | N |
| King et al. 2021 USA | Sexual health promotion | Sex education | 149 | Mixed methods | 1) A balance between autonomy and support 2) Peerness; similar, relatable and credible 3) Friendship groups & closeness to a peer educator 4) Frequency of interaction and informal diffusion beyond the classroom | Qualitative data; peer educator focus groups | 4 | High | N | N |
| Layzer et al. 2014 USA | Sexual health promotion | Sex education | 62 | Qualitative | 1) Peerness; similar, relatable and credible 2) Informal, innovative and personalised delivery methods 3) Peers as role models | Qualitative data; peer educator and peer learner focus groups | 2 | Medium | N | N |
| Layzer et al. 2017 USA | Sexual health promotion | Sex education | 1415 | Mixed methods process evaluation | 1) Peerness; similar, relatable and credible 2) Informal, innovative and personalised delivery methods 3) Peers as role models 4) Simplicity of health messages | Quantitative data; students perceptions Qualitative data; peer educator and peer learner focus groups | 4 | High | N | N |
| Mason-Jones et al. 2011 South Africa | HIV prevention | Sex education | 2339 | Quantitative | 1) A balance between autonomy and support 2) School values and broader change in school culture 3) Peerness; similar, relatable and credible | Author reflections in discussion | 3 | Medium | N | N |
| Mitchell et al. 2020 UK | Sexual health promotion | Sex education | 680 | Mixed methods | 1) Peerness; similar, relatable and credible 2) Student nominated peers and peers as role models 3) Frequency of interaction and informal diffusion beyond the classroom 4) Ratio of peer educators to peer learners | Logic model, table of mechanisms | 5 | High | Y | Y |
| Ozer 1997 USA | AIDS prevention | Sex education | 138 | Mixed methods; quantitative and classroom observations | 1) A balance between autonomy and support 2) School values and broader change in school culture 3) Peerness; similar, relatable and credible 4) Informal, innovative and personalised delivery methods | Qualitative data; classroom observations | 3 | Medium | N | Y |
| Puentes et al. 2003 USA | Family life education | Sex education | 80 | Mixed methods process evaluation | 1) A balance between autonomy and support | Qualitative data; verbal responses from peer educators and lesson observations | 2 | Medium | N | Y |
| Stephenson et al. 1998 UK | HIV/STD prevention | Sex education | 469 | Mixed methods; feasibility randomised controlled trial | 1) Informal, innovative and personalised delivery methods | Qualitative data; peer learner focus groups | 3 | Medium | N | N |
| Strange, Forrest & Oakley, 2002 UK | Sex education | Sex education | 301 | Mixed methods; Randomised controlled trial | 1) A balance between autonomy and support 2) School values and broader change in school culture 3) Informal, innovative and personalised delivery methods | Qualitative data; peer educator focus groups Quantitative data; peer educator post intervention self-report survey | 2 | Medium | N | N |
| Timol et al. 2016 South Africa | HIV/AIDS prevention | Sex education | 2904 | Quantitative; randomised controlled trial | 1) A balance between autonomy and support 2) Peerness; similar, relatable and credible 3) Simplicity of health messages | Quantitative data and author reflections/recommendations in discussion | 2 | Medium | N | N |
| Visser, 2007 South Africa | HIV/AIDS prevention | Sex education | 4086 | Mixed method; focus groups and pre-post assessment | 1) A balance between autonomy and support 2) School values and broader change in school culture 3) Informal, innovative and personalised delivery methods 4) Safe space – non-judgemental space to share experiences | Qualitative data; peer educator focus groups | 1 | Low | N | N |
