# Supplementary material for: Mechanisms of school-based peer education interventions to improve young people’s health literacy or health behaviours: A realist-informed systematic review
Source: PLoS One. 2024 May 31;19(5):e0302431. doi: 10.1371/journal.pone.0302431 (PMC11142678; doi:10.1371/journal.pone.0302431)
Supplement: Attachment — Submitted filename: PLOS ONE REVIEWER COMMENTS.pdf [file pone.0302431.s004.pdf]

The authors should be commended for the interesting topic on systematic review. I have some suggestions to improve it:

1. **Introduction**:

- Well-written novelty was clearly outlined.
- However, this is a literature review study and the authors have outlined many studies at the end, they cited one author e.g. *Many assumptions on the effectiveness of peer education for health improvement centre around 74 adolescents. For example, discussing adolescence as a time for consolidating health-related values,75 attitudes and lifestyles and making decisions about a variety of behaviors which have important 76 consequences for future health [8], as well as discussion of an increase in risk-taking behavior in 77 adolescents [9]. Many also argue that peer education is based on the rationale that peers have a 78 stronger influence on individual behavior due to the level of familiarity and trust and the comfort 79 they are able to provide [10].*
- Different referencing styles were used e.g *(Greenhalgh et al., 2007, Kaneko, 1999, O'Campo et al., 2009) and [10].*The authors must align their referencing with the ones recommended by the journal.

2. Methods

- Inclusion criteria: Authors should indicate the rationale of only including 2020 to 2023 studies.
- Furthermore, this section is not clear some studies in the reference list dated back to 1997.

3. **Results:**

- Well presented
- However authors indicated that ***studies*** *also highlighted the importance of autonomy-supportive language to motivate behavior change in peers by being encouraging and empathic without dictating what their friends should or should not do [34]*. But one author was cited in which studies??
-

4. **Discussion:** Well written

- However, authors need to discuss the implications of the findings and also create arguments based interventions of peer interventions on health outcomes might differ based on several factors context, countries , socioeconomic status, resources, etc

5. Conclusion:

- This section should summarize the important findings and outline the novelity of the study, what new (What is the novelty of the study and its scientific value?)) and what are the recommendations

6 .I have reviewed this paper from a scientific/technical perspective, and while doing so have also reviewed the language and syntax. The paper is well written.
